# Magnetic pumping model for energizing superthermal particles applied to observations of the Earth's bow shock

**DOI:** 10.1038/s41467-020-16660-4

**Published:** 2020-06-10

**Authors:** E. Lichko, J. Egedal

**Affiliations:** 1 Department of Physics, University of Wisconsin-Madison, Madison, WI USA; 2 Lunar and Planetary Laboratory, University of Arizona, Tucson, AZ USA

**Keywords:** Space physics, Plasma physics, Astrophysical plasmas

## Abstract

Energetic particle generation is an important component of a variety of astrophysical systems, from seed particle generation in shocks to the heating of the solar wind. It has been shown that magnetic pumping is an efficient mechanism for heating thermal particles, using the largest-scale magnetic fluctuations. Here we show that when magnetic pumping is extended to a spatially-varying magnetic flux tube, magnetic trapping of superthermal particles renders pumping an effective energization method for particles moving faster than the speed of the waves and naturally generates power-law distributions. We validated the theory by spacecraft observations of the strong, compressional magnetic fluctuations near the Earth’s bow shock from the Magnetospheric Multiscale mission. Given the ubiquity of magnetic fluctuations in different astrophysical systems, this mechanism has the potential to be transformative to our understanding of how the most energetic particles in the universe are generated.

## Abstract (web summary)

Energetic particle generation is an important component of a variety of astrophysical systems. Here, the authors show when magnetic pumping is extended to a spatially-varying magnetic flux tube, magnetic trapping of superthermal particles renders pumping an effective energization method for particles moving faster than the speed of the waves.

## Introduction

**Fig. 1:**
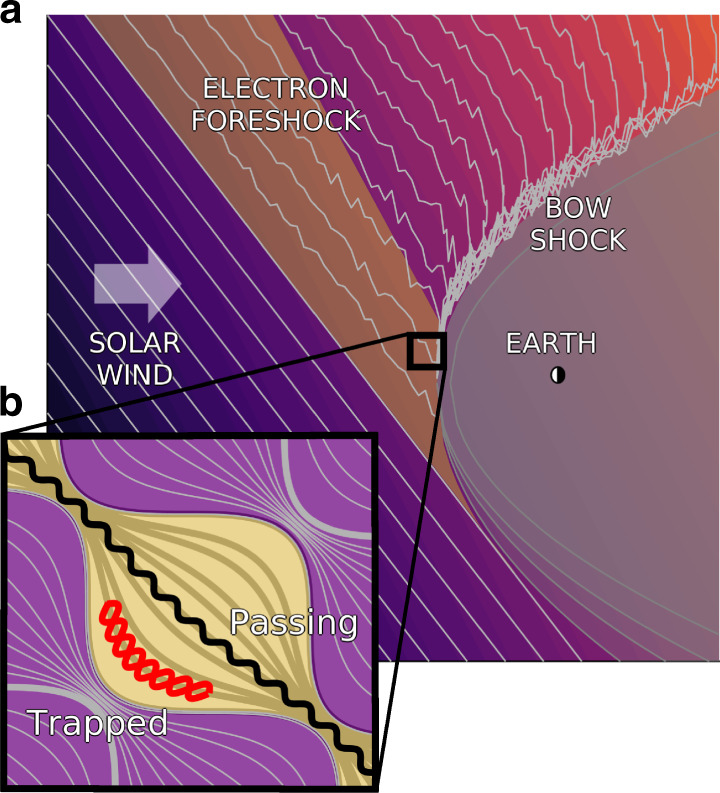
Cartoon of flux tube with spatial variation. **a** Cartoon of the incoming solar wind, including the pre-bow shock magnetic fluctuations, similar to the cartoon in Tsurutani and Rodriguez^34^. **b** The inset shows an example flux tube and a set of trapped (red) and passing (black) particle orbits.

Superthermal populations of ions and electrons are abundant in a wide variety of astrophysical systems throughout the universe, often with distributions characterized by energetic power-law tails^1,2^. However, the consensus on the physical mechanisms that energize these particles is far from settled. While it is well known that plasma can be energized by waves, most theories of wave-particle energization (excluding shocks and magnetic reconnection) are only effective for energizing particles moving at velocities close to the phase velocity of the waves^3–5^. We here present an analysis of particle energization by magnetic pumping. While previous work suggests that this mechanism is only effective up to the phase velocity of the wave^6^, we show that the addition of magnetic trapping of particle orbits renders pumping effective for heating particles moving far faster than the wave speed, an example of which is shown in Fig. 1. This mathematical treatment reveals the underlying Fermi heating mechanism consistent with the formation of energetic power-law distributions.

Diffusive shock acceleration (DSA)^7^ is a classic example of such a Fermi heating mechanism. Given an initial seed population, DSA yields power-law distributions of often relativistic electrons with Larmor radii larger or comparable to the width of the shock front. In this Letter we find that magnetic pumping in the fluctuations generated in the vicinity of Earth’s bow shock can provide a seed population of pre-energized magnetized electrons. Thus, along with mechanisms such as stochastic shock drift acceleration (SSDA)^8–10^, magnetic pumping may help address the injection problem of DSA^11,12^.

Magnetic pumping is directly related to the pressure anisotropy that naturally forms when a plasma and its magnetic field are compressed^13^. This anisotropy can be moderated by an effective scattering frequency, *ν*, caused by processes such as pitch-angle-mixing by Whistler waves^14^, or a limited confinement time of the electrons in the magnetic wells, yielding a phase delay between the perpendicular pressure, *p*_⊥_, and the flow perpendicular to the magnetic field, **u**_⊥_. Given this phase delay, when averaged over a pump cycle, mechanical work by *p*_⊥_∇_⊥_ ⋅ **u**_⊥_ then becomes finite and is the source of the energy for the magnetic pumping process. In this process energy is transferred from magnetic fluctuations to directly heat the plasma, bypassing the turbulent cascade^15–17^.

Extensive prior work, however, suggests that magnetic pumping is not effective for energizing superthermal particles. For example magnetic pumping by compressional waves, related to transit-time damping^18^, is shown to be a Landau damping process, where heating is limited to particles moving at the magnetic-field-aligned phase velocity of the wave considered, *v*_p_ = *ω*/*k*_∥_, where *ω* is the angular frequency of the wave and *k*_∥_ is the wavenumber in the direction parallel to the magnetic field^19^. In the framework of quasilinear theory Landau damping causes velocity diffusion limited to particles near the resonance velocity, *v* ~ *ω*/*k*_∥_, and is derived based on the standard procedure of integrating the plasma kinetic equations along unperturbed particle trajectories. In contrast, here we consider a standing wave geometry and apply the fast transit-time limit^20^ to retain anisotropic effects related to the full electron orbit motion.

Here we show that a quasilinear analysis with trapped electron effects and *v* ≫ *ω*/*k*_∥_ yields a velocity diffusion equation similar to that obtained by Lichko 2017^6^ for the opposite limit, *v*_∥_ ≪ *ω*/*k*_∥_. More specifically, the slowly-varying background distribution *f*_0_ is governed by a diffusion equation of the form

1 $$\frac{\partial {f}_{0}}{\partial t}=\frac{1}{{v}^{2}}\frac{\partial }{\partial v}\left({v}^{2}D\frac{\partial {f}_{0}}{\partial v}\right),\quad D=\omega {v}^{2}{\mathcal{G}}\left(\frac{\delta B}{{B}_{0}},\frac{\nu }{\omega }\right)$$

where ${\mathcal{G}}$ is independent of *v*, but is a function of *ν*/*ω*, and the magnitude of the magnetic perturbations relative to the background magnetic field, *δB*/*B*_0_. The result that *D* ∝ *ωv*^2^ is evidence of a Fermi heating process with a diffusive step size proportional to the velocity, Δ*v* ∝ *v*.

## Results

### Model of electron trapping

**Fig. 2:**
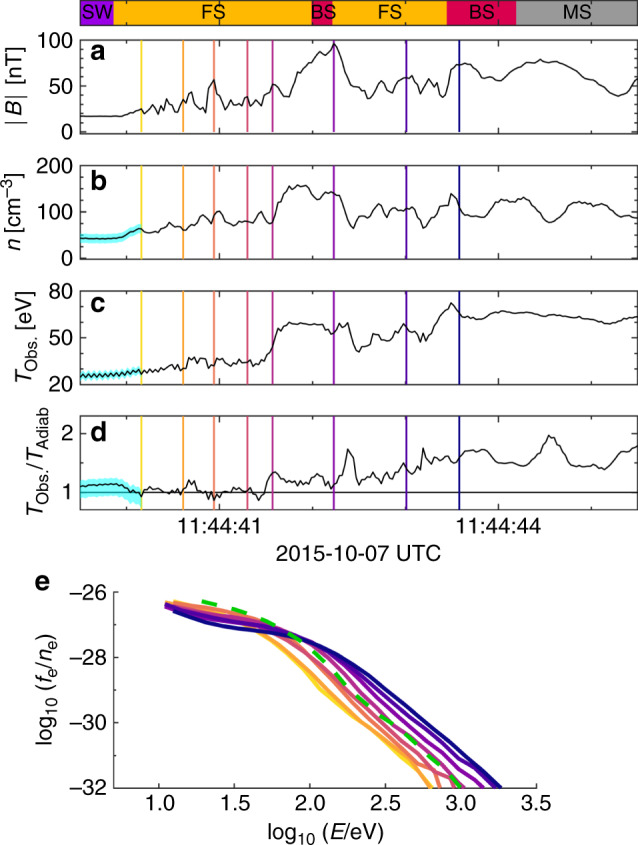
MMS data for bow shock crossing. **a** Plot of the average magnetic field from MMS1 as a function of time. The bar on the top of the plots denotes the part of the shock that the spacecraft is observing. The spacecraft is initially in the solar wind (SW) approaching the Earth's bow shock, then travels through the foreshock (FS) and the bow shock (BS) before entering the Earth's magnetosheath (MS). The part of the plot highlighted in light blue denotes the low density of the solar wind. This low density affects spacecraft performance, leading to uncertainty in the measurement of *T*_Obs._/*T*_Adiab._ in this region. **b** Plot of the density from MMS1 as a function of time. **c** Plot of the observed temperature (*T*_Obs._ = *Tr*(*P*)/(3*n*)) from MMS1 as a function of time. **d** Plot of the observed temperature as compared to the temperature expected from compressional heating alone (*T*_Adiab._ = *n*^2/3^). **e** Pitch-angle-averaged distributions at the times denoted by the colored lines in **a**–**d**. The green dashed line denotes what the final distribution at the last point should look like if it only underwent compressional heating from the initial yellow time point to the final blue time point. All of these time points are chosen to be near peaks of the magnetic fluctuations spaced upstream of this quasi-perpendicular shock front, *θ*_*Bn*_ = 83^∘^, at Alfv́nic Mach number, *M*_A_ = 6.3^27^.

Below we will outline how Eq. (1) is obtained and provide an evaluation of ${\mathcal{G}}$, a metric of the effectiveness of the pumping process. Meanwhile, we will compare these predictions to observations by the Magnetospheric Multiscale (MMS) mission in the region of the Earth’s bow shock^21^. Within the electron foreshock there are ripples, variations in the magnetic field itself, that have been shown to be a source of electron acceleration, as well as other large-amplitude magnetic-field fluctuations^22–26^, an example of which was recorded on October 7th, 2015^27^. From the time histories of ∣*B*∣, *n*, *T*_obs_, and *T*_obs_/*T*_adiabatic_, as shown in Fig. 2a–d, the temperature increase is greater than would be expected from compressional heating alone. The evolving pitch-angle-averaged distribution functions in the foreshock are shown in Fig. 2e for the times marked in Figs. 2a–d. These distribution functions demonstrate energy transfer consistent with a Fermi heating mechanism, where Δ*v* ∝ *v*, which in log–log format can be seen in the shift of the energetic power-law tails at a constant slope.

We first aim to demonstrate that the observed anisotropy is consistent with electron trapping in a standing wave perturbation and is representative of the perturbed distribution function driven during each pumping cycle. We consider the limit where the bounce time, *τ*_b_, is much smaller than the time scales associated with the waves. The instantaneous particle orbits are then described by the magnetic moment, $\mu =m{v}_{\perp }^{2}/(2B)$ and the total energy $U={\mathcal{E}}-e\Phi$, where ${\mathcal{E}}=\frac{1}{2}m{v}^{2}$, *e* is the positive electron charge, and *Φ* is the electrostatic potential. We assume electron energies larger than the electrostatic potential associated with the perturbations^28^ such that the **v** × **B** part of the Lorentz force dominates the orbit motion. Using the additional assumption that electrons are well-magnetized, with the Larmor radius smaller than the perpendicular wave length of the perturbations, *ρ*_L_ < *λ*_⊥_, for the parameters in this bow shock crossing the model is appropriate for energies 100 eV $\,\lesssim\, {\mathcal{E}}\,\lesssim\, 100$ keV.

At each time point during the magnetic pump cycle an instantaneous electron orbit is fully characterized by *μ* and ${\mathcal{E}}$. In turn, from the multiple time scale method^20,29^ the distribution must be constant along these instantaneous orbits, reducing the dimensionality of the problem.

Starting with the drift kinetic equation^30^ with pitch-angle mixing, i.e.,

2 $$\frac{df}{dt}=\nu {\mathcal{L}}f,{\mathcal{L}}=\frac{\partial }{\partial \xi }(1-{\xi }^{2})\frac{\partial }{\partial \xi },\ \xi =\frac{{v}_{\parallel }}{{({v}_{\parallel }^{2}+{v}_{\perp }^{2})}^{1/2}},$$

we change variables from $f(t,x,{v}_{\perp },{v}_{\parallel })=f(t,{\mathcal{E}},\chi )$, where *χ* = *Λ*/(*j*^2^ + *Λ*), $\Lambda =\mu {B}_{0}/{\mathcal{E}}$, and $j=J/(4vL)=1/(vL)\mathop{\int}\nolimits_{0}^{{L}_{{\rm{b}}}}{v}_{\parallel }dx$ with *L*_b_ denoting the bounce point and *L* the length of the flux-tube element. Here ${\mathcal{E}}$ and *χ* are both constant of motion variables where *χ* is representative of ${v}_{\perp }^{2}/{v}^{2}$ along the instantaneous orbits. Using these new variables and following the approach of Montag et al.^31^ and Egedal et al.^32^, we obtain an orbit-averaged form of Eq. (2)

3 $$\frac{\partial f}{\partial t}-{\left.H(t,\chi ){\mathcal{E}}\frac{\partial f}{\partial {\mathcal{E}}}\right|}_{\chi ,t}=\nu {\left\langle {\mathcal{L}}\right\rangle }_{x}f$$

where $H={\left.(2/j)(\partial j/\partial t)\right|}_{\chi }$, and the orbit average ${\left\langle (...)\right\rangle }_{x}$ is defined in the Methods subsection Orbit averaging of the Lorentz operator.

### Electron trapping in spacecraft data

**Fig. 3:**
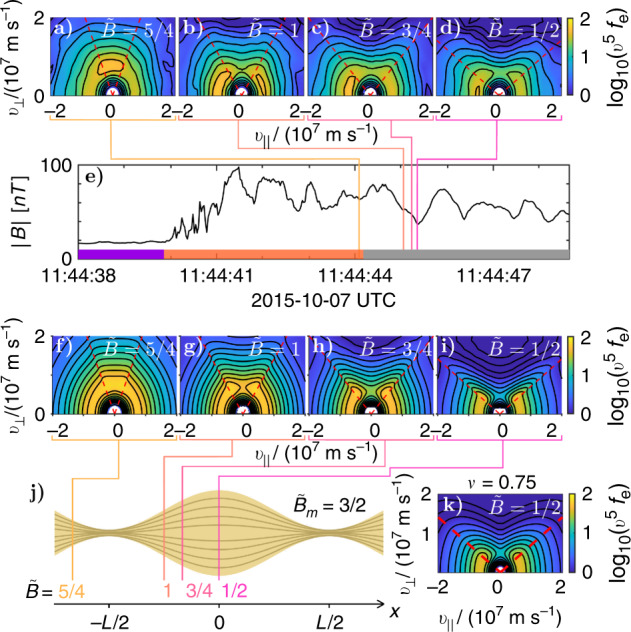
Comparison of MMS observations with model. **a**–**d** Electron distributions recorded by the Fast Plasma Investigation (FPI) instruments on MMS3 at 30 ms resolution for the rippled foreshock event reported in ref. ^27^, for the estimated points along the fluctuation where $\tilde{B}\in \{5/4,1,3/4,1/2\}$. The distributions are weighted by the factor *v*^5^ to visually enhance the anisotropic features. The trapped-passing boundary is denoted by a red dashed line. **e** Magnetic-field strength along the foreshock encounter, where the colored lines denote the times where the distributions in **a**–**d** were taken. The purple bar on the bottom denotes the part of the observation that is in the solar wind, followed by an orange bar that denotes the foreshock and bow shock, then a gray bar for the part of the observation that is in the magnetosheath. **f**–**i** Expected distribution functions computed using Eq. (3) integrated at $(\delta B/{B}_{0})\sin (\omega {t}_{0})=0.5$. The distributions are evaluated at the same $\tilde{B}$ inferred from the MMS data in **a**–**d**. In this comparison we have assumed the Taylor hypothesis^35^, that the changes in *B* recorded by the spacecraft are mainly caused by the spatial, not temporal, variations. For all electron distributions, the red dashed lines indicate the trapped/passing boundaries, characterized by ${v}_{\perp }^{2}/{v}_{\parallel }^{2}=({B}_{0}+\delta B)/B({t}_{0},x)-1$. Electrons with (*v*_∥_, *v*_⊥_) in the vicinity of these boundaries follow orbits which stagnate (*v*_∥_ ≃ 0) where ∂*B*/∂*t* is maximal, causing the orbit average of $\partial {\mathcal{E}}/\partial t=\mu (\partial B/\partial t)$ to be positive. This explains the enhanced values of *f* along the trapped-passing boundaries. **j** shows a cartoon version of the flux tube. **k** shows the theoretical distribution in **i** scattered with the Lorentz operator, ${\mathcal{L}}$, for *ν*/(*ω*/2*π*) = 0.75.

We solve Eq. (3) numerically, assuming an initial isotropic distribution and a standing wave magnetic field, $\tilde{B}(x,t)\equiv B(x,t)/{B}_{0}=1- (\delta B/{B}_{0})\sin (\omega t)\cos ({k}_{\parallel }x)$. The resultant distribution functions are shown in Fig. 3f–i for selected positions along the flux tube at a time *t*_0_ with a representative amplitude $\sin (\omega {t}_{0})(\delta B/{B}_{0})=0.5$.

Despite the idealized form of the magnetic perturbation, there is good agreement between these model distribution functions and the distribution functions observed by MMS, as shown in Fig. 3a–d, over the course of a single fluctuation of commensurate size, as shown in Fig. 3e. As documented further in the Method subsection Observations of distributions throughout the encounter, anisotropic distributions of this form are observed on all four MMS spacecraft throughout the event. To emphasize the need for pitch-angle scattering the model distributions in Fig. 3f–i were generated for *ν* = 0 and have sharper features in velocity space compared to those observed by MMS.

### Magnetic pumping model

To estimate of the aforementioned effective scattering rate we integrate the model in Eq. (3) for various values of *ν* to match the MMS observations. For example, the distribution displayed in Fig. 3k was obtained by integrating Eq. (3) with *ν*/(*ω*/2*π*) = 0.75 and provides a good match to the MMS distribution in Fig. 3d. A more detailed description of this matching process is detailed in the Methods subsection Obtaining an estimate for the effective scattering frequency, as well as ref. ^33^.

The agreement demonstrated above suggests that the model is capturing the anisotropic features of the observed distribution functions. While the inferred amount of scattering is too low for SSDA^10^ to be effective, it is near optimal for magnetic pumping and the analysis can now be extended to address the energization of the electrons over many cycles. Following the blueprint of the quasilinear method, we then separate the distribution function into the slowly-varying, isotropic background distribution, *f*_0_, and the anisotropic portion of the distribution function, *f*_1_,

4 $$f={f}_{0}(t,{\mathcal{E}})+{f}_{1}(t,{\mathcal{E}},\chi ),\quad {f}_{0}(t,{\mathcal{E}})={\left\langle f(t,{\mathcal{E}},\chi )\right\rangle }_{\chi },$$

where the pitch-angle averaging ${\left\langle (...)\right\rangle }_{\chi }$ is defined in the Method subsection Particle conservation in ${\left\langle (...)\right\rangle }_{\chi }$. To make Eq. (3) more analytically tractable, the Lorentz operator is approximated with the Krook operator, ${{\mathcal{L}}}_{{\rm{K}}}=-{C}_{{\rm{K}}}(f-{\left\langle f\right\rangle }_{\chi })$, where the factor ${C}_{{\rm{K}}}=1.15/{(\delta B/{B}_{0})}^{1.13}$ is derived in the Methods subsection Calibrating the Krook operator as a function of *δB*/*B*_0_. In addition, the anisotropic part of the distribution function, *f*_1_, as well as the other relevant anisotropic terms *H*(*t*, *χ*) and $h(t,\chi )=H-{\left\langle H\right\rangle }_{\chi }$ are Fourier transformed such that ${f}_{1}={\sum }_{n}{f}_{1}^{n}{e}^{in\omega t}$, *H* = ∑_*n*_*H*^*n*^*e*^*inωt*^, and *h* = ∑_*n*_*h*^*n*^*e*^*inωt*^.

By inserting these expansions and Eq. (4) into Eq. (3) an equation for the anisotropic part of the distribution function is found,

5 $${f}_{1}^{n}={K}_{n}{\mathcal{E}}\frac{\partial {f}_{0}}{\partial {\mathcal{E}}},\qquad {K}_{n}=\frac{{h}^{n}(-in\omega +{C}_{{\rm{K}}}\nu )}{{n}^{2}{\omega }^{2}+{({C}_{{\rm{K}}}\nu )}^{2}}.$$

Inserting *f*_1_ back into Eq. (3), an evolution equation is obtained for the slowly-varying background distribution $d{f}_{0}/dt={\langle {\langle H{f}_{1}\rangle }_{\chi }\rangle }_{t}$. More explicitly we recover Eq. (1), with

6 $${\mathcal{G}}={\mathop{\sum }\limits_{n}}\frac{{C}_{{\rm{K}}}\nu /\omega {\left\langle {\left\langle \Re ({H}^{n}{e}^{in\omega t})\Re ({h}^{n}{e}^{in\omega t})\right\rangle }_{\chi }\right\rangle }_{t}}{4{\omega }^{2}({n}^{2}+{({C}_{{\rm{K}}}\nu /\omega )}^{2})},$$

where we recall ${\mathcal{G}}$ is a measure of the energization from a single pump cycle. An approximate form of ${\mathcal{G}}$ that is easy to evaluate is given in the Methods subsection Fitting the results of ${\mathcal{G}}$.

**Fig. 4:**
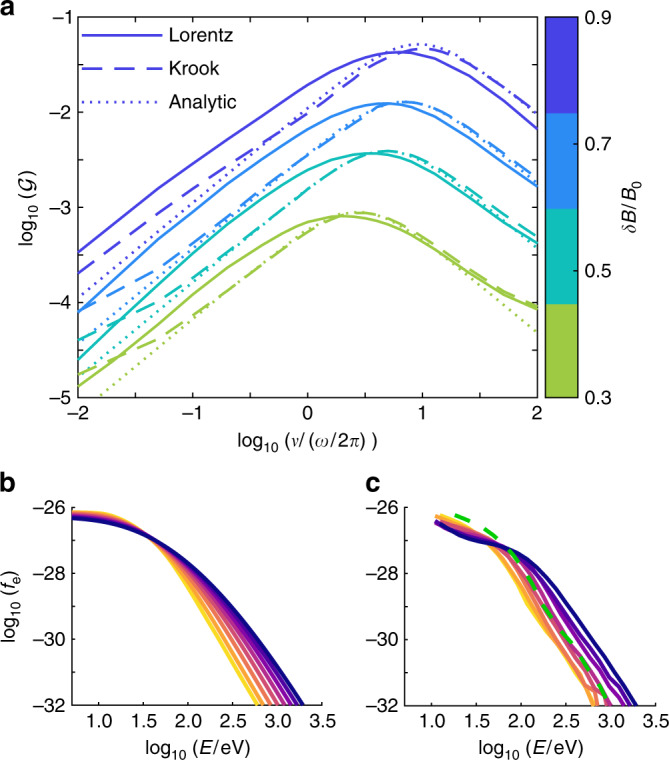
Comparison of *f*_*e*_(*v*) evolution. **a** The energization ${\mathcal{G}}$ as a function of the amplitude of a fluctuation, *δB*/*B*_0_, and the scattering frequency, *ν*, for the numerically-computed Lorentz (solid) and Krook (dashed) operators, as well as the analytical solution. **b** Combined estimate of the evolution of the distribution function from magnetic pumping and compressional heating for the points denoted in colored lines in Fig. 2a, obtained by integrating Eqs. (1) and (6) for *T* = 15 × 2*π*/*ω*, with *δB*/*B*_0_ = 0.7, and *ν*/(*ω*/2*π*) = 0.75. **c** Reproduction of Fig. 2e. The green dashed line denotes what the final distribution would look like if it only underwent compressional heating. Using these conservative parameters, ${({T}_{{\rm{Obs}}}/{T}_{{\rm{Exp}}})}_{{\rm{Theory}}}=0.83{({T}_{{\rm{Obs}}}/{T}_{{\rm{Exp}}})}_{{\rm{MMS}}}$, where most of this difference stems from the discrepancy in low energy particles.

We validate the analytical model of Eqs. (1) and (6) by integrating Eq. (3) numerically for a range of *ν* and considering a range of perturbation amplitudes, *δB*/*B*_0_. Numerical values of ${\mathcal{G}}$ in Eq. (6) are estimated through Eq. (7),

7 $$\omega \ {{\mathcal{G}}}_{{\rm{estimate}}}={\left\langle \frac{(\mathop{\int}\nolimits_{0}^{T}(\partial f/\partial t)dt)/T}{\frac{1}{{v}^{2}}\frac{\partial }{\partial v}({v}^{4}\frac{\partial f}{\partial v})}\right\rangle }_{\!\!v},$$

where *T* = 2*π*/*ω* and the numerator and denominator are found to be nearly linearly dependent functions of *v* before averaging. In Fig. 4a, the numerical model of Eq. (3) is evaluated both with the full Lorentz operator and its Krook approximation. The analytic solution in Eq. (6) (dotted) is based on the Krook model and is in good agreement with the numerical Krook result in which the efficiency of pumping increases by a factor  ~100 as *δB*/*B*_0_ is increased from 0.3 to 0.9.

### Comparison model to spacecraft data

Using the earliest spectrum (yellow) selected in Fig. 2e with a small amount of smoothing as the initial condition, we apply the model in Eqs. (1) and (6) to predict the evolution of the pitch-angle-averaged distributions recorded by MMS. Compressional heating is modeled by including $-\dot{n}/(3n)v(\partial f/\partial v)$ on the right-hand-side of Eq. (1)^6^. The result of this calculation is shown in Fig. 4b and is in good agreement with the observation from Fig. 2e, repeated for convenience in Fig. 4c. While there are some differences at lower energies, likely due to the effects of electric fields, which are neglected in our approach, the model provides a good account for the energization of electrons at large energies. The green dashed lines in Figs. 2e and 4c are the final spectra when considering compression alone, clearly underestimating the level of energization. Downstream of the shock front (for *t* > 11:44:44UT) the energization rate by pumping declines with the frequency of the fluctuations, and in combination with parallel streaming losses is consistent with a drop in the fluxes of superthermal electrons.

## Discussion

We have here presented an energization mechanism, magnetic pumping, that becomes applicable to superthermal particles, *v* ≫ *ω*/*k*, when the effects of trapping are retained. The MMS observations provide evidence that magnetic pumping has a significant role in electron energization in the region of the Earth’s bow shock. Given the potential universal applicability of the model, this could have a far-reaching impact on our understanding of electron and superthermal ion energization in many other plasma environments where particles with *v* ≫ *ω*/*k* are observed, such as the solar corona, cosmic ray generation pumped by magnetic turbulence in the interstellar medium, or possibly shocks driven by supernova explosions.

## Methods

### Finding *j* as a function of *χ*

The kinetic description applied in this work is limited to the superthermal particles characterized by speeds, *v*, sufficiently large that the Lorentz force is dominated by the magnetic term, i.e., *vB* ≫ *E*. To be more specific, in our drift kinetic analysis we are concerned with the parallel motion along the magnetic field, in general governed by forces due to the parallel electric field and the magnetic mirror force. For plasma variations of scale length *L*, the magnitude of these forces can be estimated as ∣*e*∇_∥_*Φ*∣ ≃ *T*_e_/*L* and ∣*μ*∇_∥_*B*∣ ≃ *mv*^2^*δB*/(2*B*_0_*L*), and it follows that the superthermal limit requires ${v}^{2}\gg {v}_{{\rm{t}}}^{2}{B}_{0}/(\delta B)$, where *v*_t_ is the electron thermal speed and *δB*/*B*_0_ is the normalized magnetic fluctuation amplitude.

**Fig. 5:**
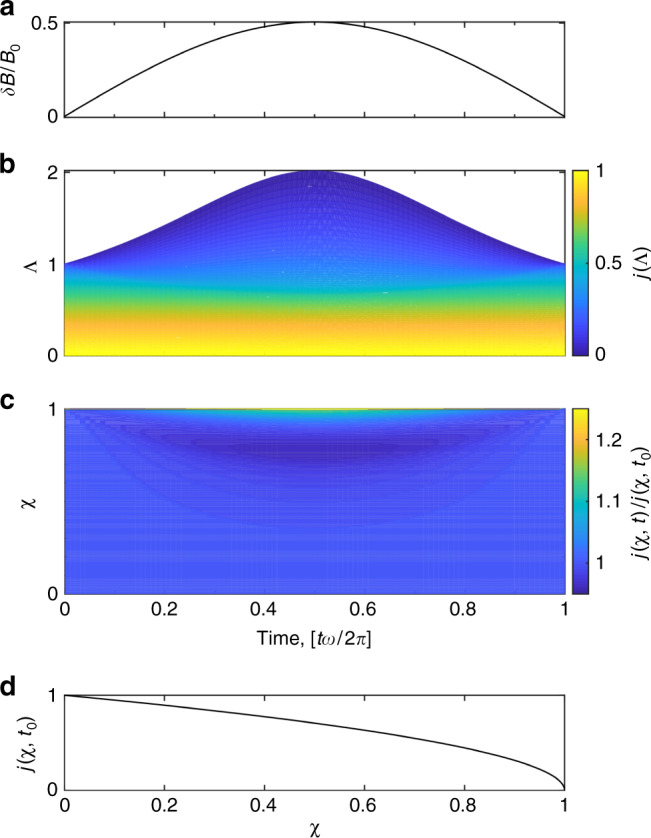
*j* as a function of invariants. **a** Plot of the maximum magnetic-field amplitude over the course of a fluctuation for a maximum magnetic field of *δB*/*B*_0_ = 0.5. **b** Plot of *j* as a function of *Λ* over the course of a fluctuation for the magnetic field in **a**. **c** Plot of *j*(*χ*, *t*) normalized by *j*(*χ*, *t*_0_) for the same fluctuation. **d** Plot of *j*(*χ*, *t*_0_).

In the superthermal limit the description of the orbit motion is significantly simplified as the value of ${v}_{\parallel }/v=\sqrt{1-\Lambda B}$ along an orbit is only dependent on $\Lambda =\mu {B}_{0}/{\mathcal{E}}$ (and independent of the electron energy ${\mathcal{E}}$ with the assumption *E*_∥_ = 0). This strongly simplifies the calculation of the second adiabatic invariant *J*(*v*, *Λ*) because *j* = *J*/(4*vL*) is then a function of only *Λ*, readily evaluated numerically for the slowly evolving magnetic perturbation considered, as illustrated in Fig. 5, where

$$j(\Lambda ,t)=\frac{1}{4L}\oint \frac{{v}_{\parallel }}{v}dl=\frac{1}{2L}\mathop{\int}\nolimits_{0}^{{L}_{{\rm{b}}}}\sqrt{1-\Lambda B(t,x)}dx.$$

Given this calculation of *j*(*Λ*, *t*) over the course of a full pump cycle we can determine *Λ*(*χ*, *t*) and in turn obtain *j*(*χ*, *t*) as shown in Fig. 5c. This function is fundamental to our analysis as it is related to the instantaneous rate of particle energization. Because *dJ*/*dt* = *d*(*vj*)/*dt* = 0 it follows that *j* *dv*/*dt* + *v* *dj*/*dt* = 0. Furthermore, as *j* = *j*(*χ*, *t*) and *dχ*/*dt* = 0, we have $dj/dt={\left.\partial j/\partial t\right|}_{\chi }$, such that ${v}^{-1}dv/dt={\left.-{j}^{-1}\partial j/\partial t\right|}_{\chi }$. We then obtain the result implicit in Eq. (3) that

$${\left.\frac{d{\mathcal{E}}}{dt}\right|}_{\chi }=-{\mathcal{E}}H\ ,\ \ {\rm{where}}\,\,H\equiv \frac{2}{j}{\left.\frac{\partial j}{\partial t}\right|}_{\chi }.$$

### Particle conservation in ${\left\langle (...)\right\rangle }_{\chi }$

Without loss of generality, in our analysis magnetic-field lines in the (*x*, *z*)-plane are characterized by the flux function Ψ, and we consider a flux-tube with constant width Δ*y* in the *y* direction. The width in the *z* direction varies as 1/*B* and is parameterized by ΔΨ. The total number of particles within our flux tube of length *L* must be conserved, i.e.,

8 $$N=\int\ f\ {d}^{3}v\ {d}^{3}x=\frac{2\pi \Delta y}{{m}^{2}}\int\ d{\mathcal{E}}\int\ d\mu \int\ d\Psi {\tau }_{{\rm{b}}}f.$$

For the point *x* = *L*/2, *B* = *B*_0_ we can evaluate ΔΨ = *B*_0_Δ*z*, where Δ*z* is the width of the flux tube at that *x* location. Furthermore, because ${\tilde{\tau }}_{{\rm{b}}}=v{\tau }_{{\rm{b}}}/(4L)$ and $d{\mathcal{E}}d\mu ={m}^{2}{v}^{3}dvd\Lambda /{B}_{0}$ we get

9 $$N=2\pi \Delta y\Delta zL\int\ {v}^{2}dv\int\ d\Lambda {\tilde{\tau }}_{{\rm{b}}}f.$$

Rewriting this in terms of our preferred variables, (*v*, *χ*) the expression becomes

10 $$N=2\pi \Delta y\Delta zL\int\ {v}^{2}dv\int\ d\chi \left(\frac{d\Lambda }{d\chi }\right){\tilde{\tau }}_{{\rm{b}}}f.$$

This motivates the averaging operator

11 $${\left\langle (...)\right\rangle }_{\chi }=\frac{\int\ d\chi \left(\frac{d\Lambda }{d\chi }\right){\tilde{\tau }}_{{\rm{b}}}(...)}{\int\ d\chi \left(\frac{d\Lambda }{d\chi }\right){\tilde{\tau }}_{{\rm{b}}}}$$

such that the pitch-angle averaged distribution becomes

12 $$F(v)={\left\langle f\right\rangle }_{\chi }.$$

In turn, the total number of particles can then be written as

13 $$N=2\pi \Delta y\Delta zL\left(\int\ d\chi \left(\frac{d\Lambda }{d\chi }\right){\tilde{\tau }}_{{\rm{b}}}\right)\int\ F(v){v}^{2}dv$$

From the form of Eq. (13) it is clear that *Fv*^2^*dv* is proportional to the number of particles in the flux tube within a differential speed interval, *dv*.

### Obtaining an estimate for the effective scattering frequency

**Fig. 6:**
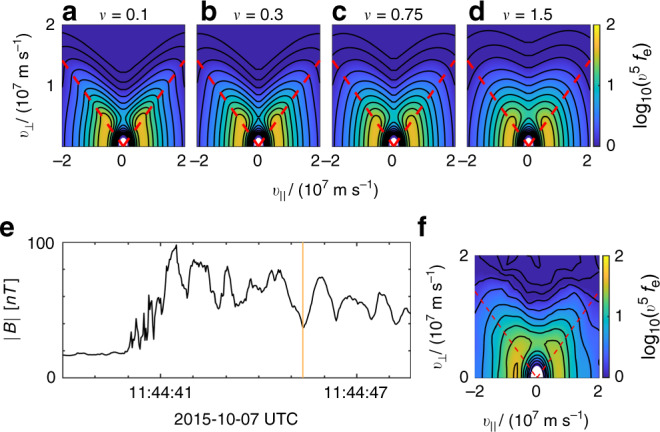
Estimating the effective scattering frequency. **a**–**d** Electron distribution functions computed using Eq. (3) integrated at $(\delta B/{B}_{0})\sin (\omega {t}_{0})=0.5$ for a range of scattering frequencies. **e** Plot of the magnetic field for the relevant bow shock crossing. **f** MMS3 observation of an electron distribution function at the orange line in **e**, where we estimate $\tilde{B}=0.5$.

We can estimate how much scattering is needed to match the spacecraft observations by comparing the MMS distributions to theoretical distributions generated by integrating Eq. (3) for a range of scattering frequencies, *ν*. By comparing the anisotropic features in the MMS distribution to the features in this set of theoretical distribution functions, we can estimate the effective scattering frequency. Based on a least-squared-fit analysis, the scattering frequency that best fit this data varies as a function of velocity, where *ν*/(*ω*/2*π*) ∈ [0.25, 1.5]. A scattering frequency within this range, *ν*/(*ω*/2*π*) = 0.75, is chosen to generate a comparison with the MMS data, where the resulting scattered distribution is shown in Fig. 3(k). A more thorough investigation into how the estimated scattering frequency varies as a function of velocity can be found in ref. ^33^, while examples of theoretical distributions scattered at a set of different rates are shown in Fig. 6.

### Observations of distributions throughout the encounter

**Fig. 7:**
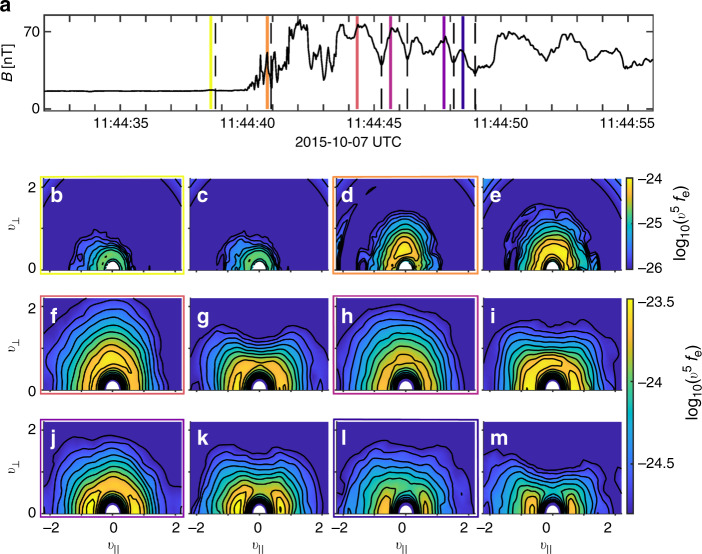
Distributions along the bow shock crossing. **a** Magnetic field observed by MMS4 along the bow shock crossing. **b**–**m** Electron distribution functions at the peaks and the corresponding valleys of magnetic fluctuations along the bow shock crossing are plotted. The distributions with the colored borders are the distributions taken at the magnetic peaks at the times denoted by the colored lines in **a**. The adjacent distributions are taken at the associated valleys denoted by the adjacent dashed lines in **a**.

In Fig. 3 of the main text we demonstrated anisotropic distribution with features matching those of the numerical model. To further demonstrate that these type of anisotropic distributions are representative for the full event, we here include in Fig. 7 electron distribution functions measured by MMS along the entire bow shock crossing. Compared to the peak and valley distributions in Fig. 3 these follow the expectations from the theory.

### Fitting the results of ${\mathcal{G}}$

**Fig. 8:**
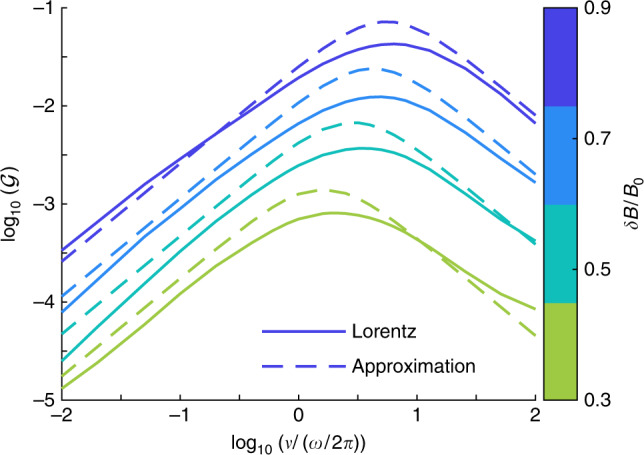
Fitting the results of ${\mathcal{G}}$. Reproduction of Lorentz distributions generated in Fig. 4 along with the results from the approximate form of ${\mathcal{G}}$.

For an easy-to-evaluate approximation of the theory, the results of Eq. (6) can be fit as

$${\mathcal{G}}\simeq 0.07\left({\left(\frac{\delta B}{{B}_{0}}\right)}^{2.6}+3{\left(\frac{\delta B}{{B}_{0}}\right)}^{5.6}\right)\frac{{C}_{{\rm{K}}}\nu /\omega }{2.3+{C}_{{\rm{K}}}^{2}{\nu }^{2}/{\omega }^{2}},$$

where ${C}_{{\rm{K}}}=1.15/{(\delta B/{B}_{0})}^{1.13}$, as discussed in the text and the last part of this methods section. This fitting, plotted alongside the results from Fig. 4a are plotted in Fig. 8. We note that this approximation for ${\mathcal{G}}$ is also valid in the limit of *δB*/*B*_0_ → 0.

### Orbit averaging of the Lorentz operator

As outlined above, our model is averaged over the fast electron orbit motion. Locally we assume that the scattering process is described by the familiar Lorentz pitch-angle scattering operator, ${\mathcal{L}}$, which is then subject to orbit averaging^20,29^. For this orbit averaging, it is convenient to express ${\mathcal{L}}$ in terms of the constant of motion variable *Λ*. Starting with the Lorentz pitch-angle scattering operator in terms of *ξ* = *v*_∥_/*v*,

14 $${\mathcal{L}}=\frac{1}{2}\frac{\partial }{\partial \xi }(1-{\xi }^{2})\frac{\partial }{\partial \xi }$$

we rewrite the scattering operator in terms of the new variables, *Λ* and ${\mathcal{E}}$

15 $${\mathcal{L}}=\frac{m{v}_{\parallel }{B}_{0}^{2}}{{{\mathcal{E}}}^{2}B}{\left.\frac{\partial }{\partial \Lambda }\right|}_{{\mathcal{E}}}\mu {v}_{\parallel }{\left.\frac{\partial }{\partial \Lambda }\right|}_{{\mathcal{E}}},\quad {v}_{\parallel }=\sqrt{2{\mathcal{E}}(1-\Lambda \tilde{B})/m}$$

which then takes the form

16 $${\mathcal{L}}=\left(\frac{2}{\tilde{B}}-3\Lambda \right){\left.\frac{\partial }{\partial \Lambda }\right|}_{{\mathcal{E}}}+\frac{2}{\tilde{B}}\Lambda (1-\Lambda \tilde{B}){\left.\frac{{\partial }^{2}}{\partial {\Lambda }^{2}}\right|}_{{\mathcal{E}}}.$$

After averaging along the spatial dimension of the flux tube we then obtain the orbit-averaged operator,

17 $$\begin{array}{l}{\left\langle {\mathcal{L}}\right\rangle }_{x}=\left(2{\left\langle \frac{1}{\tilde{B}}\right\rangle }_{x}-3\Lambda \right){\left.\frac{\partial }{\partial \Lambda }\right|}_{{\mathcal{E}}}\\ \qquad\qquad\;\;\; +\,2\Lambda \left({\left\langle \frac{1}{\tilde{B}}\right\rangle }_{x}-\Lambda \right){\left.\frac{{\partial }^{2}}{\partial {\Lambda }^{2}}\right|}_{{\mathcal{E}}},\end{array}$$

where ${\left\langle (...)\right\rangle }_{x}=1/({\tilde{\tau }}_{{\rm{b}}}L)\mathop{\int}\nolimits_{0}^{{L}_{{\rm{b}}}}dx(...)/\sqrt{1-\Lambda (B/{B}_{0})}$ is the integral over the orbits, where ${\tilde{\tau }}_{{\rm{b}}}=v{\tau }_{{\rm{b}}}/(4L)$ is the speed-normalized bounce time.

### Calibrating the Krook operator as a function of *δB*/*B*_0_

As is clear from the form of Eq. (14), the Lorentz operator describes diffusion of anisotropic features of the distribution function, *f*. Accordingly, the diffusion time scales as *τ*_D_ ∝ (*δξ*)^2^, where *δξ* is the typical scale for the anisotropic features of *f*. As the magnetic field increases, the trapped portion of the distribution function will increase commensurately, yielding larger values of *δξ*.

The Krook operator does not have this same dependence on *δξ*, as the rate of isotropization is in fact independent of *δξ*. When computing the Krook collision operator the new, scattered distribution function is formed from a linear combination of the original distribution function, and a fully isotropized version of the distribution function,

18 $${f}_{t+\Delta t}=\alpha {f}_{t}+(1-\alpha ){\left\langle {f}_{t}\right\rangle }_{\chi },$$

where $\alpha =\exp (-\nu {C}_{{\rm{K}}}\Delta t)$ determines the rate of isotropization.

To approximate the *δξ* dependency of ${\mathcal{L}}$ we have introduced the coefficient *C*_K_. From an analysis of the kinetic equation it follows that *δξ* of *f* is similar to *δξ* of *g* = *j*^2^ + *Λ*, which we used to provide a calibration for the efficiency of the Krook operator:

19 $${C}_{{\rm{K}}}=\frac{{\left\langle (g-{\left\langle g\right\rangle }_{\Lambda }){\mathcal{L}}g\right\rangle }_{\Lambda }}{{\left\langle {(g-{\left\langle g\right\rangle }_{\Lambda })}^{2}\right\rangle }_{\Lambda }}.$$

By repeating this computation for a multiple *δB*/*B*_0_ we find the result scales as

20 $${C}_{{\rm{K}}}(\delta B/{B}_{0})=1.15/{\left(\delta B/{B}_{0}\right)}^{1.13}$$

which is the result we used to compute the Krook distribution curves earlier in the paper.

## Supplementary information

Supplementary Information

## Data Availability

All relevant data are available from the corresponding author upon reasonable request. Additionally, the MMS spacecraft observations used in the production of this work can be found in the MMS Science Data Center (https://lasp.colorado.edu/mms/sdc/public/datasets/fpi/ and https://www.lasp.colorado.edu/mms/sdc/public/datasets/fields/).

## Code availability

All relevant code and Matlab routines are available from the corresponding author upon reasonable request.
