# Supplementary material for: Magnetic pumping model for energizing superthermal particles applied to observations of the Earth's bow shock
Source: Nat Commun. 2020 Jun 10;11:2942. doi: 10.1038/s41467-020-16660-4 (PMC7287107; doi:10.1038/s41467-020-16660-4)
Supplement: Supplementary file 1 — Supplementary Information [file 41467_2020_16660_MOESM1_ESM.pdf]

Reviewers' Comments:

Reviewer #1:

Remarks to the Author:

Electrons are accelerated to very high energies in space, solar and astrophysical settings, but the precise mechanism of electron acceleration remains unclear. This paper builds on earlier studies of the magnetic pumping theory that takes advantage of the ubiquitously present magnetic fluctuations/waves. In the presented work, the theory can now explain an energization of particles that are moving faster than the speed of the waves/fluctuations. The theory is also validated by the MMS observation of Earth's bow shock associated with intense fluctuations/waves. Given the ubiquity of magnetic fluctuations in different plasma environment, this mechanism has the potential to give a significant impact on our understanding of electron energization at not only Earth's bow shock but also other plasma processes in the universe.

The paper is largely theoretical and is focused on how the previous theory of magnetic pumping can be extended. However, I feel I do not qualify to comment on the theoretical part and will comment mostly on the observational part.

A key issue is that the description of the observation is too small to evaluate whether the theory has successfully reproduced the observation or not. Also, it is not adequately described how the proposed theory fits into the current picture of electron energization at Earth's bow shock; It appears to me that the presented theoretical idea is an alternative explanation of the phenomena already discussed by Katoh and Amano, ApJ, (2019) and Oka et al., ApJ, (2019). Nevertheless, this paper potentially provides an important and interesting application of the magnetic pumping mechanism to the problem of electron acceleration at shocks, and so I recommend this paper for publication in Nature Communications once the following comments are addressed.

MAJOR COMMENTS

----

1. The methodology of reproducing the observed energy spectra

A key result of this paper is in FIG.4b. However, it is not clearly described what initial condition is used to produce FIG.4b. I understand that the authors have used "the estimated parameters for the MMS event shown in Fig.2" and applied the model in Eqs. 1 and 6. However, if I understand correctly, such a procedure needs a starting spectrum from which the distribution at each colored time point is reproduced. The modeled yellow spectrum does not seem to match the observed one, and so I assume another spectrum in the far upstream was used. However, it is not described in the text.

2. Realistic conditions

The paper does not describe how some of the important, realistic effects are considered in the model. For example, the observed electron spectra may be influenced by a pre-existing non-thermal component (e.g., strahl and/or halo in the solar wind). This issue brings us back to my comment #1 on the initial condition. It may be okay if the pre-existing non-thermal component is already included in the starting spectrum. Also, the paper does not describe the possible effect of the shock potential. The paper does mention that the effects of electric fields are neglected, and I think that is okay if the authors are referring to electrostatic waves. The shock potential, however, may cause a bulk energization and may affect the spectra near 100 eV and below. Thus, the paper should at least mention if the shock potential was sufficiently small to be neglected.

3. Generality of the pumping mechanism

FIG.3 uses data from MMS3 whereas FIG.1 uses data from MMS1. Also, FIG.3 uses data after 11:44:44 whereas FIG.1 uses data before 11:44:44. Such differences raise a concern regarding the generality of the pumping mechanism. Was the anisotropic feature not observed at the time of the power-law formation? Why such a different set of data is presented?? From an inspection of the MMS quick-look plots, it appears the high-energy electron flux was enhanced before 11:44:44. Why the high-energy electron flux was higher before 11:44:44 when what appears to be the evidence of pumping mechanism (as shown in FIG.3) was detected after 11:44:44?

4. Relation to the conventional picture of electron energization.

Electron acceleration has been studied for decades by many authors. The presented paper completely ignores the vast literature on this topic. While the shock drift acceleration (SDA) has

been a key theory since 1970s, the paper does not adequately describe how the magnetic pumping differs from (or reconcile with) such a conventional standard theory. Katou and Amano (2019) extended the SDA theory to include the Fermi acceleration by waves. Oka et al. (2019) looked into more detailed process of the interaction between electrons and whistler waves, which is necessary to understand the picture by Katou and Amano (2019). They report a mirroring process which seems essentially the same process considered in the presented magnetic pumping mechanism. Thus, the presented theoretical idea may be an alternative, similar explanation of the phenomena already discussed by Katoh and Amano (2019) and Oka et al. (2019).

5. Presentation Quality

I understand there is a very large amount of theoretical work packed in this short letter. However, there is not enough amount of information needed to understand the observation. Some of the information are even incorrect. For example, very important parameters such as the shock Mach number and shock angle are not presented. Of course, some details may be written in the Johlander paper, but readers should not be asked to go back and forth between this paper and the Johlander paper, just for such a simple basic information. It is not described what time resolution was used when plotting the electron distributions. It is not clear which time period is the shock transition layer in FIG.2. The caption says all of these time points are chosen to be upstream, but my inspection of the Johlander paper revealed that reflected-gyrating ions existed even at the yellow time points, indicating that all of these time points are in the shock transition layer and not the upstream.

MINOR COMMENTS

----

0. Please add line numbers in the draft.

1. 1st paragraph, 5th line: In my understanding, the verb "heat" is used when the temperature is increasing and does not apply to acceleration to non-thermal energies. However, I think the authors are referring to non-thermal acceleration, as well, in this paragraph.

2. 1st paragraph, lines 6-10: This is may be misleading, because I think there are many non-resonant wave-particle interactions that can be important for particle acceleration. A famous example is the diffusive shock acceleration.

3. 4th paragraph: If p_perp grad_perp dot u_perp is the source of energy, can this be verified in the MMS data? How can this be distinguished from the motional electric field which is the key source of energy in the well-known Shock Drift Acceleration process?

4. 6th paragraph: In my understanding, ripples are generated in the shock transition layer, not in the upstream. Please see the references 13-17.

5. FIG.1: This figure is misleading because it would give an impression that the waves responsible for magnetic pumping already exists in the solar wind. However, the whistler waves at the shock can well be generated at the shock front. The wave intensity in the pristine solar wind is much less than that in the shock transition layer and magnetosheath.

6. p.3, right column, 2nd line from the top: (f-i) instead of (a-h)?

7. p.3, right column, 8th line from the top: (a-d) instead of (f-i)?

8. p.3, right column, 10th line from the top: (e) instead of (j)?

Reviewer #2:

Remarks to the Author:

Review of the paper Lichko and Egedal, Magnetic pumping model for energizing superhtermal particles, applied to in-situ particle observations, submitted to Nature Communications

The paper is on very important topic in space physics with possible impacts also to astrophysical plasma as particle energization is a fundamental plasma process in the universe. The paper is very nicely written, topic is concise and figures are of excellent quality and informative. It has potential to be published in Nature Communications.

Main comments:

At parts the paper is difficult to follow for a reader who is not very familiar with the topic. Theory part is very detailed, but those parts that are related to comparison with the observations could be discussed in more detailed. In addition, some results shown in the figures are discussed very lightly in the text (or not at all) and the related discussion should be extended.

How does Figures 2 and 3 relate? It appears that Figure 2 is a zoom-in of Figure 3? And how do the times selected in Figures 2 and 3 relate? I.e., Figure 2 has times for which the pitch-angle-averaged distributions are calculated selected from the solar wind before the bow shock to magnetosheath, while in Figure 3 they seem to have been selected, except the first one, within one “ripple”/flux tube. And does then heating demonstrated in Figure 3 feature overall heating when the spacecraft moves from solar wind into the magnetosheath, while Figure 3 shows the heating that is occurring within one of the ripples/flux tube? Also Figure 1 shows this “ripple”/flux tube in the foreshock region, while the data and times taken in Figure 3 seems to be from the magnetosheath only? Are these ripple/flux tube structures where electrons get pumped to high energies occurring both in the foreshock regions and in the magnetosheath? Earlier in the text it was mentioned that they occur in the upstream of the bow shock. These would require some clarification

Page 3: Figure 3 shows the results for v/(omega/2pi)=0.75. Is this the parameter for which the agreement was the best? How sensitive the matching is for the scattering parameter and is the excellent agreement unique or does several scattering parameter values give very good agreement as well? (e.g., does the agreement worsen quickly if v is changed slightly)?

Detailed comments:

Page 2: It should be explained in more detail how Fermi heating can be seen from Fig 2e

Figure 2: Is the bow shock transition visible from the figure? It could be marked there if so. Are periodic small fluctuations from spacecraft spin?

Page 2: Where is the anisotropy visible?

The logic in selecting times in Figure 2 should be explained. Are they at the peak field of the successive flux tubes/ripples close to the bow shock as in Fig 1

Authors should check that all variables are explained in the text. Also, some parameters could be explained a bit more, e.g. scattering parameter seems very important parameter for this study and its significance could be briefly introduced when appearing for the first time in the text.

Page 3, below Equation (3): Authors could refer here that the details of the averaging parameter is given in the methods part.

Page 3: B tilde should be explained in the text and why these B-values were selected for investigation

Page 3 and 4: While theory is explained in detail, observations and comparison to computed distributions should be discussed in more detail, e.g., different key features and how they relate at different locations in tube.

Fig 4a) The results related to different magnetic field values (i.e. different colored lines in the plot) are not discussed in the text. Is Delta_B same as delta_B in the text and does it have importance for the results? Also, why Krook approximation is clearly in better agreement with the analytical solution than full Lorentz operation solution?

It could be emphasized somewhere in the end of the paper that the green curve in figures 2e and 4c where only compressional heating is considered underestimates clearly the heating in the studied event.

Reviewer #3:

Remarks to the Author:

This manuscript presents the first in situ observational evidence that magnetic pumping may play a fundamental role in generating superthermal particles with power-law distributions. Power-law particle distributions are commonly observed in space and astrophysics and are generally attributed to high energy, transient phenomena such as shocks and magnetic reconnection. Magnetic pumping

is a ubiquitous process caused by fluctuations of the magnetic field modulus coupled with particle scattering, which are generic features of most turbulent plasmas in the universe. The ubiquity of the physical process and the in situ evidence of magnetic pumping suggest it is a viable mechanism for generating energetic particles throughout the universe. The importance of this work is evident and publication in Nature is well justified. However, a few, mostly minor, points detailed below, if addressed, would further improve the quality of the manuscript.

1) The introductory material could be improved. First, the magnetic pumping process is not clearly defined in the manuscript. Given the broad readership of Nature, it would be very helpful to provide a simple, two or three sentence description. Also, the general context of the importance of power-law distributions and energetic particles should be expanded beyond the two sentences currently devoted to them in the introduction to better motivate and contextualize the results. For example, the standard processes for generating high energy particles (shocks and reconnection) are not mentioned in this context, nor is a reason provided that an additional mechanism like magnetic pumping is required to explain observations.

2) The reference for transit-time damping should include Barnes' original paper, Barnes, Phys Fluids, 9, 1483 (1966).

3) The statement "In the framework of quasilinear theory Landau damping causes velocity diffusion limited to particles near the resonance velocity" is only accurate for a monochromatic wave. Voitenko & Pierrard, Sol. Phys., 288, 369 (2013) demonstrate that quasilinear diffusion due to a spectrum kinetic Alfven waves leads to the development of extended distribution function plateaus, extending to a few times vA.

4) The choice to show modeled contours of f in figure 3f-i with nu = 0 seems odd, since in this case, magnetic pumping is reversible. It would be helpful to state a reason for this choice in the manuscript.

5) The statement "The resultant distribution functions are shown in Fig. 3(a-h)" refers to the wrong panels. It should be 3(f-i), unless I have misunderstood what is plotted in 3(a-d).

6) That fig 3k "provides an excellent match" to the in situ data in 3d is too vague. It is later stated that a least-squares fit was done to match the data. The resulting chi squared for the fit would be helpful to supply so that the reader can better evaluate the fit quality.

7) The coefficient C_K is not defined in the body of the manuscript. It should either be defined in the body, or a note should be added to see the methods section for the definition.

8) Figure 4b was produced using N_fluctuations = 15, but the explicit dependence on number of fluctuations does not appear anywhere else in the manuscript and this point should be at least briefly discussed in the manuscript. For instance, how sensitive is f to N_fluctuations, and is N_fluctuations = 15 a realistic value for the observation?

9) In producing figure 4b, a model of compressional heating is also included. To better evaluate the relative importance of pumping and compressional heating, it would be helpful to provide a the ratio of the two heating mechanisms. It would be valuable to know if the ratio is comparable to T_Obs/T_Adiabatic shown in figure 2d for the in situ data.

We thank the referees for their detailed reviews, which have helped us improve the manuscript. Below are our point by point response to the referee comments, and all comments have been addressed by modifications to the manuscript (as highlighted in blue).

Reviewer #1 (Remarks to the Author):

Electrons are accelerated to very high energies in space, solar and astrophysical settings, but the precise mechanism of electron acceleration remains unclear. This paper builds on earlier studies of the magnetic pumping theory that takes advantage of the ubiquitously present magnetic fluctuations/waves. In the presented work, the theory can now explain an energization of particles that are moving faster than the speed of the waves/fluctuations. The theory is also validated by the MMS observation of Earth's bow shock associated with intense fluctuations/waves. Given the ubiquity of magnetic fluctuations in different plasma environment, this mechanism has the potential to give a significant impact on our understanding of electron energization at not only Earth's bow shock but also other plasma processes in the universe.

The paper is largely theoretical and is focused on how the previous theory of magnetic pumping can be extended. However, I feel I do not qualify to comment on the theoretical part and will comment mostly on the observational part.

A key issue is that the description of the observation is too small to evaluate whether the theory has successfully reproduced the observation or not. Also, it is not adequately described how the proposed theory fits into the current picture of electron energization at Earth's bow shock; It appears to me that the presented theoretical idea is an alternative explanation of the phenomena already discussed by Katoh and Amano, ApJ, (2019) and Oka et al., ApJ, (2019). Nevertheless, this paper potentially provides an important and interesting application of the magnetic pumping mechanism to the problem of electron acceleration at shocks, and so I recommend this paper for publication in Nature Communications once the following comments are addressed.

MAJOR COMMENTS
----

1. The methodology of reproducing the observed energy spectra

A key result of this paper is in FIG.4b. However, it is not clearly described what initial condition is used to produce FIG.4b. I understand that the authors have used "the estimated parameters for the MMS event shown in Fig.2" and applied the model in Eqs. 1 and 6. However, if I understand correctly, such a procedure needs a starting spectrum from which the distribution at each colored time point is reproduced. The modeled yellow spectrum does not seem to match the observed one, and so I assume another spectrum in the far upstream was used. However, it is not described in the text.

Reply: The initial condition in Fig. 4b is indeed a slightly smoothed version of the “yellow” spectrum recorded by MMS. This has been clarified in the manuscript.

2. Realistic conditions

The paper does not describe how some of the important, realistic effects are considered in the model. For example, the observed electron spectra may be influenced by a pre-existing non-thermal component (e.g., strahl and/or halo in the solar wind). This issue brings us back to my comment #1 on the initial condition. It may be okay if the pre-existing non-thermal component is already included in the starting spectrum. Also, the paper does not describe the possible effect of the shock potential. The paper does mention that the effects of electric fields are neglected, and I think that is okay if the authors are referring to electrostatic waves. The shock potential, however, may cause a bulk energization and may affect the spectra near 100 eV and below. Thus, the paper should at least mention if the shock potential was sufficiently small to be neglected.

Reply: As addressed in (1), it is correct that the initial distribution includes a halo component, which is part of our initial distribution. Meanwhile, in the MMS distributions for the energies considered we see no evidence that a parallel streaming strahl is part of the heating process. This has been clarified in the manuscript.

Our model applies the drift-kinetic framework that is applicable to well-magnetized electrons with Larmor radii below the perpendicular wave length of the magnetic fluctuation. For such well-magnetized electrons the shock potential will drive ExB flows which do not cause any energization, and will not impact the electron distribution function when expressed in our constant-of-motion variables. Furthermore, the cross-shock potential may yield a small potential drop over the parallel wavelength of a single magnetic perturbation, which by standard kinetic arguments must be smaller than the electron temperature. Thus, in the present framework, we do not see any mechanism by which the cross-shock potential can influence our pumping model in the superthermal limit. This has been clarified in the manuscript.

3. Generality of the pumping mechanism

FIG.3 uses data from MMS3 whereas FIG.1 uses data from MMS1. Also, FIG.3 uses data after 11:44:44 whereas FIG.1 uses data before 11:44:44. Such differences raise a concern regarding the generality of the pumping mechanism. Was the anisotropic feature not observed at the time of the power-law formation? Why such a different set of data is presented?? From an inspection of the MMS quick-look plots, it appears the high-energy electron flux was enhanced before 11:44:44. Why the high-energy electron flux was higher before 11:44:44 when what appears to be the evidence of pumping mechanism (as shown in FIG.3) was detected after 11:44:44?

Reply: We observe similar anisotropic features on all four spacecraft throughout the event. The data in Fig. 3 was selected because the magnetic field displayed a simple sinusoidal form, well suited for showcasing the model. To demonstrate that the same form of the anisotropy is

representative throughout the event, in the methods section we now include an additional figure with MMS data demonstrating this point.

The anisotropic features are a required ingredient in the pumping model, but the rate of heating by pumping is also highly sensitive to the pumping frequency (see Eqs. 1 and 6 in the manuscript) which is much reduced at the later time after the main shock has passed. In combination with parallel streaming losses (not included in the model) the reduced heating rate is consistent with the reduction in the flux of high-energy electrons observed at the later time. This has been clarified in the manuscript.

4. Relation to the conventional picture of electron energization.

Electron acceleration has been studied for decades by many authors. The presented paper completely ignores the vast literature on this topic. While the shock drift acceleration (SDA) has been a key theory since 1970s, the paper does not adequately describe how the magnetic pumping differs from (or reconcile with) such a conventional standard theory. Katou and Amano (2019) extended the SDA theory to include the Fermi acceleration by waves. Oka et al. (2019) looked into more detailed process of the interaction between electrons and whistler waves, which is necessary to understand the picture by Katou and Amano (2019). They report a mirroring process which seems essentially the same process considered in the presented magnetic pumping mechanism. Thus, the presented theoretical idea may be an alternative, similar explanation of the phenomena already discussed by Katoh and Amano (2019) and Oka et al. (2019).

Reply: We just noticed the Feb 2020 PRL which includes the authors mentioned above (Amano, Katou and Oka). In their stochastic shock drift acceleration (SSDA) model the electrons are confined in the parallel direction by strong scattering off of whistler waves, and heated by a parallel DC-electric field. While some of the ingredients are similar, this SSDA model has fundamental differences compared to ours. For example, in our model the electrons are confined through magnetic trapping and not by strong pitch angle diffusion. In fact, the high levels of pitch angle diffusion required for the SSDA model would render our model inefficient. Furthermore, in magnetic pumping the particles are energized by the drift kinetic term “magnetic moment X dB/dt” which is very different from the parallel DC electric field invoked in the SSDA model.

For the event presented here the pitch angle scattering is inferred directly from the anisotropic features in the electron distributions. Thus, we are very confident in the inferred pitch angle rate (normalized to the fluctuation rate) of \mu/(\omega/2\pi)~0.75. That level is near optimal for magnetic pumping but is far too small to confine the electrons in the parallel direction (along the magnetic field), as required by the SSDA model. These observations suggest that heating by pumping and SSDA is unlikely to overlap (pumping requires moderate scattering rates whereas SSDA requires strong scattering).

Meanwhile, like the SSDA model our pumping framework applies to well magnetized electrons and is therefore important to the injection problem of the more standard Diffusive Shock Acceleration models.

These issues have been clarified in the revised manuscript.

5. Presentation Quality

I understand there is a very large amount of theoretical work packed in this short letter. However, there is not enough amount of information needed to understand the observation. Some of the information are even incorrect. For example, very important parameters such as the shock Mach number and shock angle are not presented. Of course, some details may be written in the Johlander paper, but readers should not be asked to go back and forth between this paper and the Johlander paper, just for such a simple basic information. It is not described what time resolution was used when plotting the electron distributions. It is not clear which time period is the shock transition layer in FIG.2. The caption says all of these time points are chosen to be upstream, but my inspection of the Johlander paper revealed that reflected-gyrating ions existed even at the yellow time points, indicating that all of these time points are in the shock transition layer and not the upstream.

Reply: We have added in the additional information requested, including the Alfv\'en Mach number and shock angle from the Johlander paper. All of the electron distribution function data shown were taken from a single measurement, which for these spacecraft is in a 30 ms window. We have added in notations to Fig. 2 and 3 to denote where the different parts of the shock start and stop.

MINOR COMMENTS

----

0. Please add line numbers in the draft.

Reply: We have added in line numbers to the draft.

1. 1st paragraph, 5th line: In my understanding, the verb "heat" is used when the temperature is increasing and does not apply to acceleration to non-thermal energies. However, I think the authors are referring to non-thermal acceleration, as well, in this paragraph.

Reply: Thanks, we have replaced it with “heating and non-thermal energization”.

2. 1st paragraph, lines 6-10: This is may be misleading, because I think there are many non-resonant wave-particle interactions that can be important for particle acceleration. A famous example is the diffusive shock acceleration.

Reply: Thanks, we have revised the paragraph.

3. 4th paragraph: If p_perp grad_perp dot u_perp is the source of energy, can this be verified in the MMS data? How can this be distinguished from the motional electric field which is the key source of energy in the well-known Shock Drift Acceleration process?

We agree that magnetic pumping does not preclude other energization processes, and have changed the manuscript to clarify this point. That said, besides compressional heating, we see no evidence in the data of other proposed heating mechanisms in this event (note again that SSDA is excluded by the low pitch angle scattering rates inferred). The direct evidence for pumping provided here is the electron pressure anisotropy and the inferred level of scattering: as our theoretical calculation shows these ingredients lead directly to the energization of the plasma, where the source of the energy is the mechanical energy provided by the time average of p_perp grad_perp dot u_perp.

It is possible that through a large statistical analysis of several MMS shock crossings that the time average of p_perp grad_perp dot u_perp can be inferred directly, but that is beyond the scope of the present manuscript.

4. 6th paragraph: In my understanding, ripples are generated in the shock transition layer, not in the upstream. Please see the references 13-17.

Reply: Thanks, this mistake has been corrected.

5. FIG.1: This figure is misleading because it would give an impression that the waves responsible for magnetic pumping already exists in the solar wind. However, the whistler waves at the shock can well be generated at the shock front. The wave intensity in the pristine solar wind is much less than that in the shock transition layer and magnetosheath.

Reply: Thanks, we have modified the figure to reflect that we are considering waves/turbulence generated by the shock front.

6. p.3, right column, 2nd line from the top: (f-i) instead of (a-h)?
7. p.3, right column, 8th line from the top: (a-d) instead of (f-i)?
8. p.3, right column, 10th line from the top: (e) instead of (j)?

Reply: Our apologies – we forgot to change the labels in the text when we changed the figure. We have updated the text to reflect the actual figure labels. Thank you!

Reviewer #2 (Remarks to the Author):

Review of the paper Lichko and Egedal, Magnetic pumping model for energizing superhtermal particles, applied to in-situ particle observations, submitted to Nature Communications

The paper is on very important topic in space physics with possible impacts also to astrophysical plasma as particle energization is a fundamental plasma process in the universe. The paper is very nicely written, topic is concise and figures are of excellent quality and informative. It has potential to be published in Nature Communications.

Main comments:
At parts the paper is difficult to follow for a reader who is not very familiar with the topic. Theory part is very detailed, but those parts that are related to comparison with the observations could be discussed in more detailed. In addition, some results shown in the figures are discussed very lightly in the text (or not at all) and the related discussion should be extended.

How does Figures 2 and 3 relate? It appears that Figure 2 is a zoom-in of Figure 3? And how do the times selected in Figures 2 and 3 relate? I.e., Figure 2 has times for which the pitch-angle-averaged distributions are calculated selected from the solar wind before the bow shock to magnetosheath, while in Figure 3 they seem to have been selected, except the first one, within one “ripple”/flux tube. And does then heating demonstrated in Figure 3 feature overall heating when the spacecraft moves from solar wind into the magnetosheath, while Figure 3 shows the heating that is occurring within one of the ripples/flux tube? Also Figure 1 shows this “ripple”/flux tube in the foreshock region, while the data and times taken in Figure 3 seems to be from the magnetosheath only? Are these ripple/flux tube structures where electrons get pumped to high energies occurring both in the foreshock regions and in the magnetosheath? Earlier in the text it was mentioned that they occur inthe upstream of the bow shock. These would require some clarification

Reply: Similar issues were raised above by review 1 and we have clarified that anisotropic features consistent with those in Fig.3 are observed at all times where strong magnetic perturbations are observed. As clarified in the figures, the magnetic ripples are observed both in the foreshock and at lower frequency in the magnetosheath. In the magnetosheath heating by pumping will still occur but at lower rates (due to the reduced frequencies).

We expect that the heating/energization in the magnetosheath is offset by parallel heat-losses. While this has been clarified in the manuscript a detailed analysis of the energy balance in the magnetosheath is beyond the scope of the present letter.

Page 3: Figure 3 shows the results for v/(omega/2pi)=0.75. Is this the parameter for which the agreement was the best? How sensitive the matching is for the scattering parameter and is the

excellent agreement unique or does several scattering parameter values give very good agreement as well? (e.g., does the agreement worsen quickly if v is changed slightly)?

Reply: In the methods section we now include distributions computed as several different values of \nu/(\omega/2\pi). Form these it follows that the scattering rate is determined with an accuracy of about 50%. A more detailed analysis in given in Lichko's PhD thesis, which shows that the quoted value is representative for the range of energies considered. This has been clarified in the manuscript.

Page 2: It should be explained in more detail how Fermi heating can be seen from Fig 2e

Reply: We have added some clarifying details to the description of Fig. 2 e).

Figure 2: Is the bow shock transition visible from the figure? It could be marked there if so. Are periodic small fluctuations from spacecraft spin?

Reply: We have added in labels for the different parts of the shock which are visible in this time range, consistent with the Johlander paper.

The fast ripples were not physical, but were related to changing biasing applied in the electron detector. We have eliminated them for clarity.

Page 2: Where is the anisotropy visible?

The anisotropy is discussed in connection with Fig. 3.

The logic in selecting times in Figure 2 should be explained. Are they at the peak field of the successive flux tubes/ripples close to the bow shock as in Fig 1.

Reply: They are at the peak magnetic field points distributed along the bow shock crossing. Because there is a natural variation in the distribution function over the course of a fluctuation, it is important to choose the same point within in the fluctuation so that the change in energy that is seen is due to pumping, and not the natural variation within a fluctuation. The peaks and valleys are the easiest parts of the fluctuation to identify. We have added some addition text explaining our choice of timepoints in the caption of Fig. 2.

Authors should check that all variables are explained in the text. Also, some parameters could be explained a bit more, e.g. scattering parameter seems very important parameter for this study and its significance could be briefly introduced when appearing for the first time in the text.

Reply: We have now added in explanations of variables that were not previously defined in the text and discussed the scattering parameter in more detail.

Page 3, below Equation (3): Authors could refer here that the details of the averaging parameter is given in the methods part.

Reply: We have added this into the text.

Page 3: B tilde should be explained in the text and why these B-values were selected for investigation

Reply: B tilde is the normalized magnetic field and we have clarified that the selected value is representative for the observations.

Page 3 and 4: While theory is explained in detail, observations and comparison to computed distributions should be discussed in more detail, e.g., different key features and how they relate at different locations in tube.

Reply: We have extended the discussion of this in the caption of Fig. 3.

Fig 4a) The results related to different magnetic field values (i.e. different colored lines in the plot) are not discussed in the text. Is Delta_B same as delta_B in the text and does it have importance for the results? Also, why Krook approximation is clearly in better agreement with the analytical solution than full Lorentz operation solution?

Reply: The Krook numerical and analytical solutions are in good agreement as they both use the Krook scattering model in place of the more complicated Lorentz model. This has been clarified in the manuscript.

Thanks – Delta_B should be delta_B/B_0, and the legend of the plot has been updated.

It could be emphasized somewhere in the end of the paper that the green curve in figures 2e and 4c where only compressional heating is considered underestimates clearly the heating in the studied event.

Reply: Thanks, we have added some additional text where Fig. 4 is discussed.

Reviewer #3 (Remarks to the Author):

This manuscript presents the first in situ observational evidence that magnetic pumping may play a fundamental role in generating superthermal particles with power-law distributions. Power-law particle distributions are commonly observed in space and astrophysics and are generally attributed to high energy, transient phenomena such as shocks and magnetic reconnection. Magnetic pumping is a ubiquitous process caused by fluctuations of the magnetic field modulus coupled with particle scattering, which are generic features of most turbulent plasmas in the universe. The ubiquity of the physical process and the in situ evidence of magnetic pumping suggest it is a viable mechanism for generating energetic particles throughout the universe. The importance of this work is evident and publication in Nature is well justified. However, a few, mostly minor, points detailed below, if addressed, would further improve the quality of the manuscript.

1) The introductory material could be improved. First, the magnetic pumping process is not clearly defined in the manuscript. Given the broad readership of Nature, it would be very helpful to provide a simple, two or three sentence description. Also, the general context of the importance of power-law distributions and energetic particles should be expanded beyond the two sentences currently devoted to them in the introduction to better motivate and contextualize the results. For example, the standard processes for generating high energy particles (shocks and reconnection) are not mentioned in this context, nor is a reason provided that an additional mechanism like magnetic pumping is required to explain observations.

Reply: Thanks, we agree that our discussion was incomplete and we now provide more details on other Fermi heating models which yield power-law distributions.

2) The reference for transit-time damping should include Barnes' original paper, Barnes, Phys Fluids, 9, 1483 (1966).

Reply: We have added this reference.

3) The statement "In the framework of quasilinear theory Landau damping causes velocity diffusion limited to particles near the resonance velocity" is only accurate for a monochromatic wave. Voitenko & Pierrard, Sol. Phys., 288, 369 (2013) demonstrate that quasilinear diffusion due to a spectrum kinetic Alfven waves leads to the development of extended distribution function plateaus, extending to a few times vA.

Reply: We have updated the text to be consistent with the referee comment, however in the context of electrons, flattening of the distribution extending a few times vA, is still very limited compared to what is needed to generate power-laws.

4) The choice to show modeled contours of f in figure 3f-i with nu = 0 seems odd, since in this case, magnetic pumping is reversible. It would be helpful to state a reason for this choice in the manuscript.

Reply: We choose nu=0 in order to show that this yields very fine structures in f and that a finite value of nu is needed to account for the smoother features seen in the MMS data. This has been clarified in the manuscript.

5) The statement "The resultant distribution functions are shown in Fig. 3(a-h)" refers to the wrong panels. It should be 3(f-i), unless I have misunderstood what is plotted in 3(a-d).

Reply: Our apologies – we forgot to change the labels in the text when we changed the figure. We have updated the text to reflect the actual figure labels. Thank you!

6) That fig 3k “provides an excellent match” to the in situ data in 3d is too vague. It is later stated that a least-squares fit was done to match the data. The resulting chi squared for the fit would be helpful to supply so that the reader can better evaluate the fit quality.

Reply: Similar issues were raised in review 2 and in the methods section we now provide theoretical distributions computed for four different scattering rates. This provides some detail on the sensitivity of the distribution against the pitch angle scattering. More details on the least square fitting is available in Lichko’s PhD thesis, now included in the list of references.

7) The coefficient C_K is not defined in the body of the manuscript. It should either be defined in the body, or a note should be added to see the methods section for the definition.

Reply: C_K is now defined in the main text and a note pointing to the methods section is added.

8) Figure 4b was produced using N_fluctuations = 15, but the explicit dependence on number of fluctuations does not appear anywhere else in the manuscript and this point should be at least briefly discussed in the manuscript. For instance, how sensitive is f to N_fluctuations, and is N_fluctuations = 15 a realistic value for the observation?

Reply: The number N of fluctuations dictates the time interval to be considered when integrating Eqs 1 and 6 (where time is normalized by the pump frequency). It is not directly known from the fluctuations what N should be, but N~15 appears to be a conservative number consistent with the observations. This has been clarified in the manuscript.

9) In producing figure 4b, a model of compressional heating is also included. To better evaluate the relative importance of pumping and compressional heating, it would be helpful to provide a the ratio of the two heating mechanisms. It would be valuable to know if the ratio is comparable to T_Obs/T_Adiabatic shown in figure 2d for the in situ data.

Reply: We now provide this value in the caption of Fig. 4.

REVIEWERS' COMMENTS:

Reviewer #1 (Remarks to the Author):

The authors have addressed all of my concerns and I now recommend this paper for publication in Nature Communications. I'm sorry again for this delayed review report.

Reviewer #2 (Remarks to the Author):

This paper has been improved considerably. The topic and text are still very theoretical, but observations are described in much

more detail + several parts that were unclear have been now explained. I’m happy to suggest the paper for publication. Only a few small points I noticed:

Line 84: Could be that it slipped my eye, but cannot find the definition for v (nu).

Lines 123-127: This sentence is difficult to read and could be reformulated.

Figure 3: >Was the validity of Taylor hypothesis checked or only assumed that it applies?

Reviewer #3 (Remarks to the Author):

The authors have well addressed my original concerns, and I now find the content of the manuscript fully appropriate for publication in Nature Communications. However, there are a few very minor

issues related to several figures in the paper that I do recommend are addressed prior to publication.

1) Figure 1 is no longer referenced anywhere in the body of the manuscript.

2) Figure 2 panels b-d now have cyan highlighted regions. The meaning of the highlighting is not stated anywhere, and it is unclear why panel a would not also be similarly highlighted.

3) Figure 7 is referenced on line 460, but it seems as though this line should refer to figure 6.

4) Figures 7 and 8 are not referenced anywhere in the body of the methods section. It seems that figures 7 and 8 should be referenced in the paragraphs beginning on lines 463 and 472, respectively.

We would like to thank the reviewers for the time and effort that they spent revising this article. The final result is much improved as a result of your efforts! Below we have included our point by point responses to the comments.

Reviewer #1 (Remarks to the Author):

The authors have addressed all of my concerns and I now recommend this paper for publication in Nature Communications. I'm sorry again for this delayed review report.

Reviewer #2 (Remarks to the Author):

This paper has been improved considerably. The topic and text are still very theoretical, but observations are described in much
more detail + several parts that were unclear have been now explained. I'm happy to suggest the paper for publication. Only a few small points I noticed:

Line 84: Could be that it slipped my eye, but cannot find the definition for v (nu).

I believe that we define nu on Line 52 - it is the effective scattering frequency.

Lines 123-127: This sentence is difficult to read and could be reformulated.

We have reworded the sentence to be more clear. It now reads "Using the additional assumption that electrons are well-magnetized, with the Larmor radius smaller than the perpendicular wave length of the perturbations, $\rho_\mathrm{L}<\lambda_{\perp}$, for the parameters in this bow shock crossing the model is appropriate for energies 100eV$\lesssim{\cal E}\lesssim 100$keV."

Figure 3: >Was the validity of Taylor hypothesis checked or only assumed that it applies?

We assumed that the Taylor hypothesis applies – we have changed 'applied the Taylor hypothesis' to 'assumed the Taylor hypothesis' to reflect this.

Reviewer #3 (Remarks to the Author):

The authors have well addressed my original concerns, and I now find the content of the manuscript fully appropriate for publication in Nature Communications. However, there are a few very minor issues related to several figures in the paper that I do recommend are addressed prior to publication.

1) Figure 1 is no longer referenced anywhere in the body of the manuscript.

We have added back a reference to Fig. 1 in the first paragraph – thank you!

2) Figure 2 panels b-d now have cyan highlighted regions. The meaning of the highlighting is not stated anywhere, and it is unclear why panel a would not also be similarly highlighted.

Our apologies – we now explain the cyan highlighted regions in the figure legend.

3) Figure 7 is referenced on line 460, but it seems as though this line should refer to figure 6.

Thank you for pointing that out – we have fixed the figure reference.

4) Figures 7 and 8 are not referenced anywhere in the body of the methods section. It seems that figures 7 and 8 should be referenced in the paragraphs beginning on lines 463 and 472, respectively.

We have fixed these figure references, thank you!
